# Agri-ecological dataset from vegetation surveys on organic legume fields in Tuscany, Italy

**DOI:** 10.1016/j.dib.2024.110306

**Published:** 2024-03-11

**Authors:** Anna-Lena Vollheyde, Christina von Haaren

**Affiliations:** Institute of Environmental Planning, Leibniz University Hannover, Herrenhäuser Straße 2, 30419 Hannover, Germany

**Keywords:** Flora, Weeds, Biodiversity, Sustainable agriculture, Pulses

## Abstract

Legumes are becoming increasingly important regarding the transformation of food consumption and production systems towards more sustainability. Apart from supporting and production services, legumes can also enhance biodiversity in agroecosystems. In this dataset, we present results from vegetation surveys of 244 samplings on 32 lentil and chickpea fields of five organic farms in Tuscany, Italy. Centre and edge zones of the fields were surveyed separately. Additionally, the dataset provides a comprehensive summary of the associated management practices applied to the respective fields as well as a characterisation of the site conditions through soil texture, organic matter, local weather data during the legume growing period and the diversity of the field's landscape contexts. This additional extensive characterisation of the management system and environment allows the data to be used for a variety of multivariate analysis on biodiversity and agroecosystems.

Specifications Table

**Table:** 

Subject	Biodiversity, Agronomy and Crop Science
Specific subject area	Flora biodiversity in relation to associated agricultural practices, soil, climate and landscape context
Data format	Raw, (Semi-) Analysed
Type of data	.xlsx file (data tables and metadata)
Data collection	Vegetation surveys were done in lentil and chickpea fields of organic farms in Tuscany in summer 2022 and 2023. Wild flora was mapped in 1×2m sample-plots following drilled lines with a randomised sampling. Two strata (edge and centre) were sampled with four repetitions each. Associated management practices of the fields were retrieved via post-hoc farmer's surveys. Soil and weather data were deduced from Tuscan pedological map and local weather stations. The diversity of the landscape context was analysed through Shannon Index of semi-natural habitats based on High Nature Value farmland data, CORINE Land cover and Copernicus high resolution geodata in a 1 km buffer around the fields.
Data source location	Legume fields of organic farms in Tuscany, Italy
Data accessibility	Repository name: Forschungsdaten-Repositorium der Leibniz Universität Hannover Data identification number: https://doi.org/10.25835/ffwtmdgv Direct URL to data: https://data.uni-hannover.de/dataset/dataset-flora-diversity-and-agri-environmental-information-organic-legume-fields-tuscany

## 1 Value of the Data


 

• This dataset encompasses the results of vegetation surveys in chickpea and lentil fields of organic farms in Tuscany, Italy, along with corresponding data on agricultural management practices, soil characteristics, weather conditions, and contextual variables.
• Vegetation surveys were conducted with a stratified sampling design, whereby edge and centre zones are differentiated.
• The detailed and comprehensive provision, especially also of the associated agricultural management operations and environmental characterisations of the fields, allows other researchers to utilise the dataset not only for univariate analysis but also for multivariate analysis of agroecosystem processes such as plant-environment or biodiversity-management relationships and thus deducing leverage points in agro-biodiversity conservation.

## 2 Background

Pulses are recognised as a vital plant-based protein source for humans. They are getting greater importance for shifting current food consumption and production systems toward greater sustainability [1,2]. Amongst other advantages, such as nitrogen fixation ability, carbon sequestration and higher drought resistance, growing legumes can also benefit biodiversity. In this context, the conservation of genetic diversity (local landraces) and diversification of crop rotations and landscapes are predominantly mentioned [2,3]. There are only few data about flora diversity under different management regimes and in different landscape contexts. However, this information is needed for designing sustainable management schemes for pulses cultivation as well as for agri-environmental remuneration on the basis of projected biodiversity.

## 3 Data Description

**Fig. 1:**
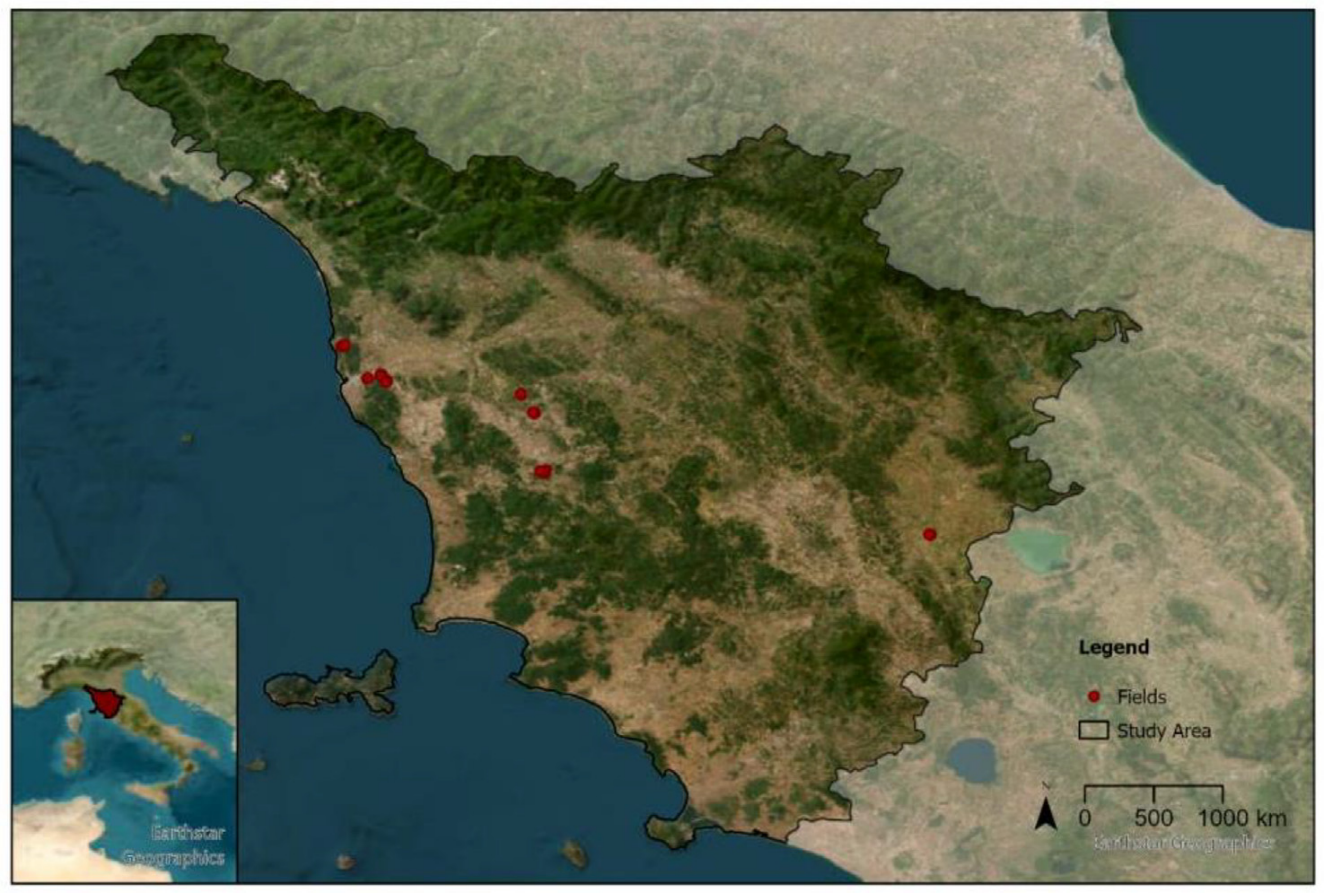
Allocation of the sampled fields (points can overlap).

Here we describe a biodiversity dataset by means of wild flora, together with associated agricultural management practices, soil, weather, and context variables sampled on organic lentil and chickpea fields in Tuscany, Italy [4]. The sampling was done on 32 fields from five farms, including one research farm. The farms are located along a West-East axis in the regions Pisa, Livorno, Peccioli, Pomerance and Torrita di Siena (see Fig. 1). Due to a repetitive sampling design (see section Materials and Methods), the dataset consists of 244 samplings.

**Fig. 2:**
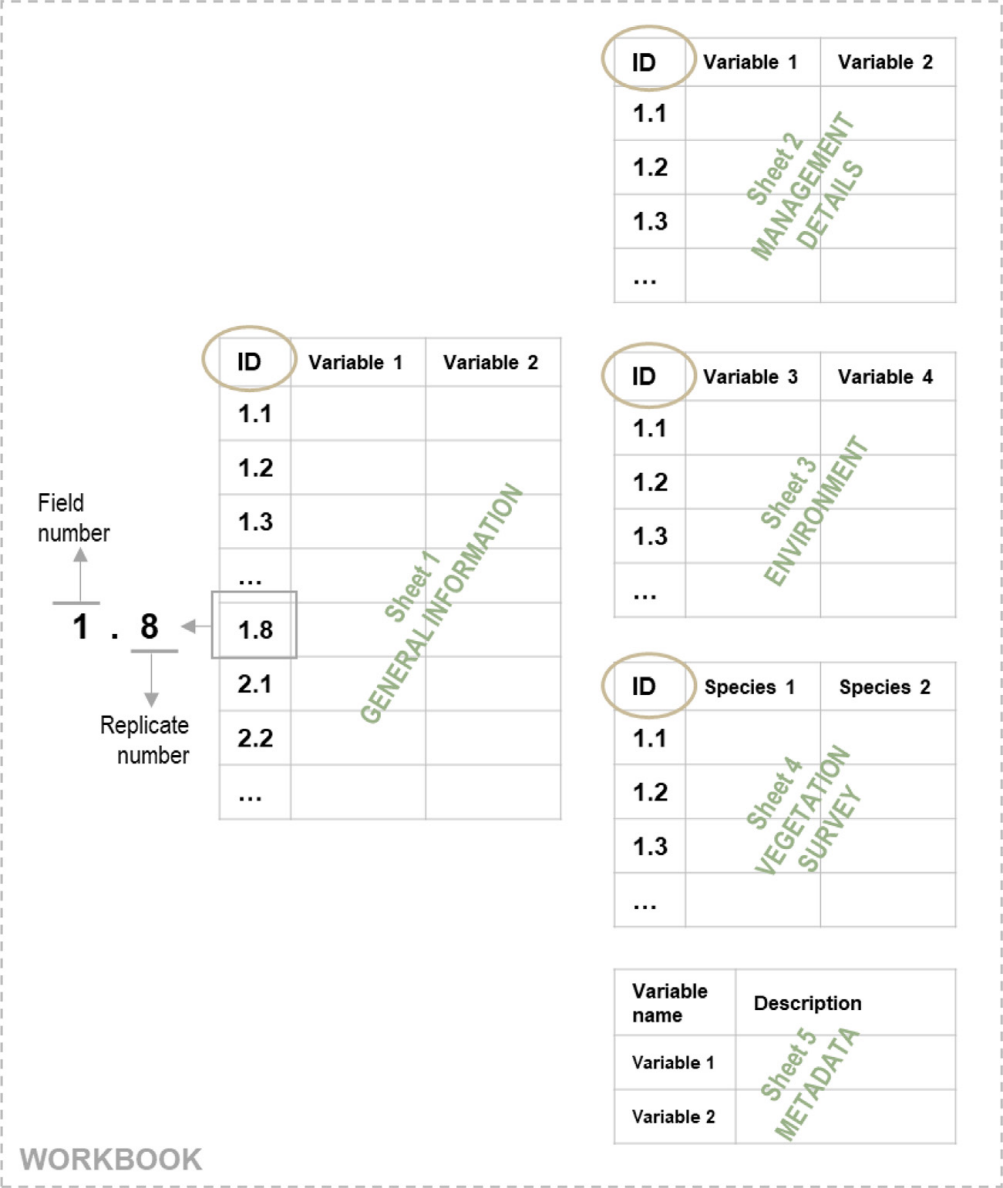
Structure of the dataset.

The data are provided in one workbook file with a total of five sheets: four sheets contain the core dataset, and an additional sheet with metadata describing the given variables. Each database entry in the four core data tables represents data from one sample-plot and has a unique identifier (“ID”) composed of two numbers separated by a point. The pre-decimal integer is the unique field identifier. The decimal number represents the replicate number of the respective field. All sheets, except the metadata table, can be joined via the ID field (see Fig. 2).

The core dataset contains general information, management details, environmental data and the vegetation survey results in separate sheets which are named accordingly. General information is provided by means of the e.g. sampling year, field size or the allocation of the sample plot (edge/centre). In the management details, a comprehensive overview of the applied cultivation practices of each field are compiled. These include, for example: growing period, sowing specifications, crop rotation, tillage and weeding practices, fertilisation details, a proxy for management intensity and legume yield. The sheet “environment” provides information on soil texture, organic matter, weather during the growing period and landscape context. Lastly, the results of the vegetation survey are provided in another sheet whereby the cover (%) of the respective species is given in the cells, as well as in the last two columns, total species richness and Shannon Index per sample-plot are provided.

## 4 Experimental Design, Materials and Methods

The data sampling process can be divided into i) on-field sampling as well as post-hoc data acquisition through ii) management surveys and iii) analysis of weather, soil, and landscape context geodata. Statistical analysis was conducted in the R environment (version 4.0.3). Geospatial analysis was done using ArcGIS Pro 3.1.2.

### 4.1 On-field sampling and vegetation survey

**Fig. 3:**
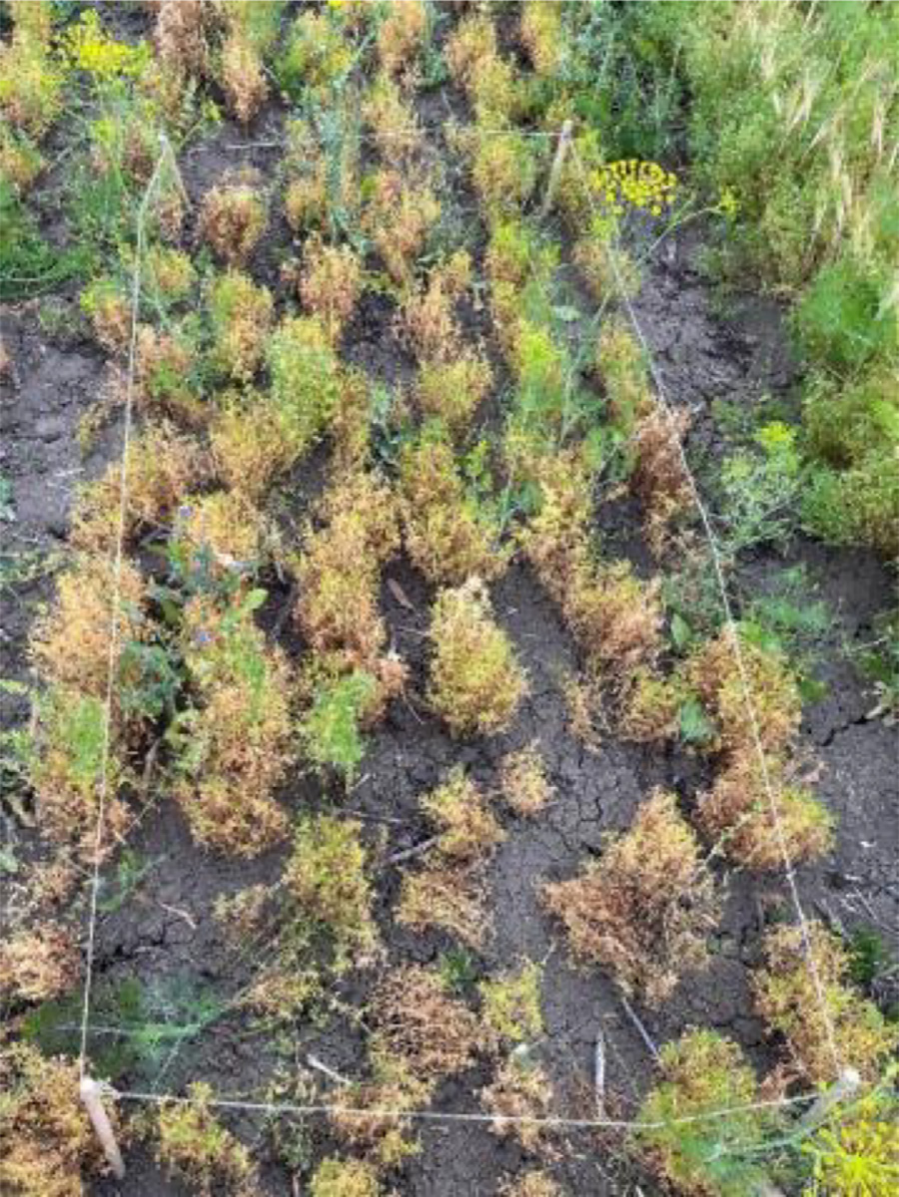
Placement of the sample-plots (picture: Vollheyde 2022).

We conducted a wild flora mapping in 1×2m sample-plots. In each field, we applied a stratified sampling method to account for edge effects and the intensity gradient one usually finds on fields [5]. The strata were the two field zones: centre and edge. As edge, we defined the first 3m from the field boundary. In each zone, four replicates were sampled, resulting in eight samplings per field. To reduce the proximity of the sample-plots and the strata during the random placement, we sprawled out from the field middle for the centre strata and circuited the whole field for the edge strata. All sample-plots were randomly placed within the respective zone following the drilled lines (see Fig. 3). An exception had to be made on three fields on a research farm, where the fields were too small to divide into edge and centre strata. Here we only sampled four replicates in the exact middle of the field.

The sampling was done in June/July 2022 and 2023. We conducted a full vegetation survey of wild flora following the Braun-Blanquet method and additionally mapped other parameters related to the legume crop. A full list of parameters mapped on-field in each sample plot is given in Table 1. For a higher precision, the degrees of coverages were not mapping according to pre-defined classes, but freely estimated on an integer scale. If small species were present with a very low abundance (e.g. only one individual of *Anagallis arvensis* L.) in a sample-plot, a true coverage could not realistically be estimated. In these cases, a coverage of <1% was noted, which in further analysis was always transformed and treated as 0.5%. Flora species nomenclature was post-hoc harmonised according to Euro+Med PlantBase [6]. Species richness and Shannon Index of wild flora of each sample-plot were calculated using the vegan package [7].

**Table 1:** Parameters mapped during fieldwork.

Parameter	Explanation
Legume growth stage	BBCH scale, according to [8]
Allocation of the plot	centre/ edge
Total plant cover (crops and wild flora)	Freely estimated in %
Cover of each crop	Freely estimated in %
Total cover of all wild flora	Freely estimated in %
Full wild flora species list including individual coverage	Coverage freely estimated in %

### 4.2 Management survey

The applied management details were retrieved post-hoc from the farmers, or their agronomists respectively, and were collected for each sampled field individually. The information was retrieved via a written survey. Each filled survey sheet was checked for missing information and inconsistencies in given answers. In any of these cases, the respective answers were clarified in a follow-up online meeting or phone call. The requested management information encompasses details about: field size, cultivars, crop rotation and duration, sowing density, general management intensity, yield as well as applied tillage, fertilisation and weed management regimes.

### 4.3 Analysis of weather, soil, and landscape context geodata

Weather data during the legume growing phase were retrieved post-hoc. We defined the legume growing phase as the period from the day of legume sowing until the day of harvest. For those periods, we extracted the total precipitation (in mm) and the average temperature (in°C) from the nearest weather station to the respective field/farm (for our cases retrieved through [9], [10], [11]).

Based on aerial pictures and on-field recorded coordinates of the fields’ centre and edges, all sampled fields were digitalised using ArcGIS Pro 3.1.2. By overlaying the Tuscan pedological maps [12] with the digitalised fields, we obtained mean values for sand, silt, and clay content (in %) as well as organic matter content (in %) per field.

**Fig. 4:**
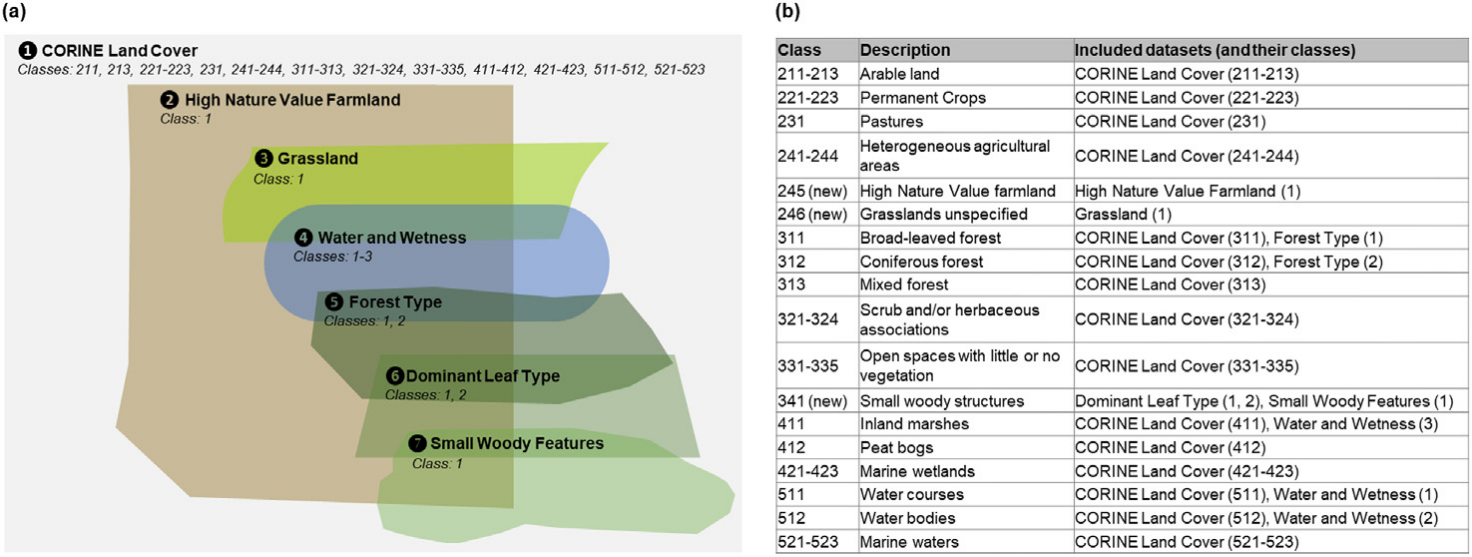
Approach to deduce the diversity of semi-natural habitats in the fields’ context. The referred datasets are Copernicus Land Monitoring Service data (vers. 2018) (CORINE Land Cover [14], Grassland [16], Water and Wetness [17], Forest Type [18], Dominant Leaf Type [19], Small Woody Features [20]) as well as High Nature Value farmland data, (vers. 2017) [13] based on [15]. (a) Workflow to retrieve a harmonised geodata set of semi-natural habitats, with ascending numbers the respective datasets bring more detail to the merged dataset. Datasets were overlaid, erased and unioned in that order. (b) Overview of the final created semi-natural habitat classes and the respective included classes of the different datasets. The classes are an extension of the CORINE Land Cover classes.

Additionally, landscape diversity by means of the Shannon diversity Index of the semi-natural habitats was analysed within a buffer zone of 1 km around each field. To identify semi-natural habitats, we combined High Nature Value farmland data [13], a selection of CORINE Land cover classes [14] that aligns to the classes related to selected for High Nature Value farmland identification in [15] and Copernicus Land Monitoring Service high resolution data (Grassland [16], Water and Wetness [17], Forest Type [18], Dominant Leaf Type [19], Small Woody Features [20]). The geodata were intersected with another in the order of their degree of detail they can add to the previous data (see Fig. 4). After that the Shannon Index of semi-natural habitats within the buffer zone of each field was calculated with the vegan package [7].

## Limitations

With 244 samplings (including repetitions) from 32 fields from five farms, the sample size of the dataset is relatively small. Moreover, except for the precondition to conduct organic agriculture, the farms were not chosen based on pre-defined criteria, such representativeness of certain practices or site conditions. Farms were included based on their willingness to participate in the study.

## Ethics Statement

The authors confirm that they have read and followed the ethical requirements for publication in Data in Brief. The work does not involve animal experiments or any data collected from social media platforms. All farmers were informed about the purpose of this study and gave consent for participation and permission for visiting and surveying their fields. All collected data are anonymized.

## CRediT authorship contribution statement

**Anna-Lena Vollheyde:** Conceptualization, Methodology, Investigation, Data curation, Writing – original draft, Writing – review & editing. **Christina von Haaren:** Conceptualization, Methodology, Writing – review & editing, Supervision, Funding acquisition.

## Data Availability

Dataset on wild flora diversity and associated agri-environmental information on organic legume fields in Tuscany (Italy) (Original data) (Forschungsdaten-Repositorium der Leibniz Universität Hannover).

## References

[1] Kwasny T., Dobernig K., Riefler P. (2022). Towards reduced meat consumption: a systematic literature review of intervention effectiveness, 2001-2019. Appetite.

[2] Stagnari F., Maggio A., Galieni A., Pisante M. (2017). Multiple benefits of legumes for agriculture sustainability: an overview. Chem. Biol. Technol. Agric..

[3] Everwand G., Cass S., Dauber J., Williams M., Stout J.C., Murphy-Bokern D., Stoddard F.L., Watson C.A. (2017). Legumes in Cropping Systems.

[4] A. Vollheyde, C. von Haaren, Dataset on wild flora diversity and associated agri-environmental information on organic legume fields in Tuscany (Italy), 2024. 10.25835/ffwtmdgv. PMC10957370 38524837

[5] Romero A., Chamorro L., Sans F.X. (2008). Weed diversity in crop edges and inner fields of organic and conventional dryland winter cereal crops in NE Spain. Agric. Ecosyst. Environ..

[6] Euro+Med 2006+, Euro+Med PlantBase - the information resource for Euro-Mediterranean plant diversity., continuously updated. http://www.europlusmed.org/ (accessed 14 December 2023).

[7] J. Oksanen, G.L. Simpson, F.G. Blanchet, R. Kindt, P. Legendre, P.R. Minchin, R. O'Hara, P. Solymos, M.H.H. Stevens, E. Szoecs, H. Wagner, M. Barbour, M. Bedward, B. Bolker, D. Borcard, G. Carvalho, M. Chirico, M. De Caceres, S. Durand, H.B. Antoniazi Evangelista, R. FitzJohn, M. Friendly, B. Furneaux, G. Hannigan, M.O. Hill, L. Lahti, D. McGlinn, M.-H. Ouellette, E. Ribeiro Cunha, T. Smith, A. Stier, C.J. Ter Braak, J. Weedon, vegan: Community Ecology Package, 2022. https://CRAN.R-project.org/package=vegan.

[8] U. Meier, Growth stages of mono-and dicotyledonous plants: BBCH Monograph, second ed., 2001.

[9] ILMETEO.it, IL METEO Meteo e previsioni del tempo in Italia, 2023. https://www.ilmeteo.it/ (accessed 30 November 2023).

[10] Visual Crossing Corporation, Weather Data & Weather API | Visual Crossing, 2023. https://www.visualcrossing.com/ (accessed 30 November 2023).

[11] Meteostat, The Weather's Record Keeper | Meteostat, 2023. https://meteostat.net/en/ (accessed 30 November 2023).

[12] Regione Toscana, DB Pedologico della Regione Toscana 2015.

[13] European Environment Agency, High nature value farmland 2012 accounting version, Nov. 2017.

[14] European Union, Copernicus land monitoring service 2021, European Environment Agency, CORINE Land Cover 2018.

[15] Paracchini M., Petersen J., Hoogeveen Y., Bamps C., Burfield I., van Swaay C. (2008).

[16] European Union (2018).

[17] European Union, Copernicus land monitoring service 2020, European Environment Agency, Water and Wetness 2018.

[18] European Union, Copernicus land monitoring service 2021, European Environment Agency, Forest Type 2018.

[19] European Union, Copernicus land monitoring service 2021, European Environment Agency, Dominant Leaf Type 2018.

[20] European Union, Copernicus land monitoring service 2021, European Environment Agency, Small Woody Features 2018.

